# “Does he need help or can he help himself?” Preschool children’s expectations about others’ instrumental helping versus self-helping

**DOI:** 10.3389/fpsyg.2014.00430

**Published:** 2014-05-12

**Authors:** Sunae Kim, Beate Sodian, Markus Paulus

**Affiliations:** Department of Psychology, Ludwig Maximilian University of MunichMunich, Germany

**Keywords:** social cognition, children, instrumental helping, reasoning, prosociality

## Abstract

In the present study, we investigated a total of fifty-one 3.5-, 4.5-, and 5.5-year-old children’s expectations about another person’s helping behaviors. We asked children to complete a story in which one person failed to complete his goal (e.g., because an object was misplaced or put out of his reach) while the other person observed the event. We asked whether the children expected the other person to help the protagonist or whether they expected the protagonist to help himself. Children of 3.5 years expected the other person to provide help in the majority of trials. In contrast, the older children were equally likely to predict that the other person would help the protagonist or the protagonist would help himself.

## INTRODUCTION

Recent research has shown that very early in development children engage in a variety of prosocial behaviors such as helping, sharing, and comforting (for reviews see Brownell, 2013; Tomasello and Vaish, 2013; Paulus, 2014). Already in infancy children are willing to help others complete a simple action related goal even in the absence of verbal request (Warneken and Tomasello, 2006; Dunfield and Kuhlmeier, 2010; Svetlova et al., 2010; Dunfield et al., 2011; Paulus et al., 2013). For example, 1-year-old children readily helped an adult, who was unable to complete a task because an object was out of her reach, by bringing the object to her (Warneken and Tomasello, 2006), and by 24 months children provided help even when the other did not notice the accident (Warneken, 2013). Three-year-old children provided help specific to goal completion, offering a different object more suitable for others’ goal completion rather than a requested object (Martin and Olson, 2013). Interestingly, children provided help even to non-human agents (Kenward and Gredebäck, 2013) suggesting that the inclination to help might be very strong in children. Such prosocial behavioral tendencies are supposed to support the development of stable social relationships (e.g., Eisenberg et al., 1996).

A potentially equally important prerequisite for the engagement in successful social interactions is knowledge about which prosocial behaviors can be expected from others. These expectations further guide one’s future interactions with others, at times creating tensions and conflicts if others’ behaviors are not consistent with the expectations. It is thus important to understand how children develop expectations of others’ prosocial behavior and identify the situations in which these behaviors do or do not occur.

An early study examining children’s expectations about others’ prosocial behavior comes from Berndt (1981). He showed that children of ages 5–10 indeed expected others to display prosocial behaviors, but equally toward friends vs. non-friends. Recent findings show that expectations about others’ prosocial behaviors are present early in development and become more sophisticated with increasing age. Even 15-month-old infants seem to expect someone to share equally with others (e.g., Sloane et al., 2012). Children of ages 4–5 years, but not 3 years, expected others to share more with friends than disliked peers (Paulus and Moore, 2014).

Although these findings deepen our understanding of how children conceive of others’ sharing, only little is known about their expectations of others’ instrumental helping. In light of recent findings that the different types of prosocial behavior (i.e., helping, sharing, comforting) do not relate to each other (e.g., Dunfield and Kuhlmeier, 2013) and that even different neurophysiological activations are related to instrumental helping vs. comforting (Paulus et al., 2013), we should be cautious about generalizing findings from children’s expectations about others’ sharing to their expectations about others’ helping. That is, children’s prosocial behaviors in terms of helping beyond toddlerhood are not entirely understood. Only one recent study examined children’s reasoning about others’ (non)helping. Sierksma et al. (2013) found that children between the ages of 8–13 years approved someone’s refusal to help when helping is costly to a helper and a potential helpee’s need of help is low. Nevertheless, because this study focused on school-aged children, it remains an open question how preschool children reason about others’ instrumental helping. The present study aimed to examine preschool aged children’s expectations about others’ helping behaviors when helping involved low cost to the helper. We chose the low cost helping scenarios in order to maximally facilitate children’s reasoning about helping. Young children’s helping emerges earlier in low-cost helping situations than costly helping situations (Svetlova et al., 2010). Given that we were interested in the early emergence of reasoning about others’ helping behaviors, we presented low-cost helping scenarios to children.

To this end, we assessed children’s expectations about others’ helping behaviors in a third party context. We presented children with six scenarios in which one person was in need of help to complete his/her simple action related goals and the other person could offer help. The helping scenarios were similar to tasks used in prior research in which children faced another person who was in need of help in completing his/her simple action related goals (e.g., Warneken and Tomasello, 2006). We were interested in children’s naturally occurring expectations of others’ helping behaviors – whether a potential helpee would receive help by the other person or solve his problem without help. Therefore, we asked children open-ended questions to predict what would happen in the given scenarios. Given infants’ strong tendency for instrumental helping (e.g., Warneken and Tomasello, 2006) we expected that our youngest age group would respond that the helpee would receive help from the other person. Moreover, as children’s prosocial behaviors are explicitly encouraged by parents and teachers their expectations of others’ helping may become increasingly strong with age. Alternatively, older children may consider other factors such as someone’s action capability to complete his goals himself and underlying intentions for an incomplete action (e.g., being genuinely in need of help or being playful or tricky). Children’s understanding of others’ action goals and intentions (Barresi and Moore, 1996; Paulus and Moore, 2011; Paulus et al., 2011) and their theory of mind (see Perner, 1991) develop during preschool years. In addition, children’s increasing development of autonomy may contribute to their expectations about others’ helping behaviors. As children gain independence and autonomy they are likely to enjoy carrying out actions on their own. This may lead them to expect others to be equally autonomous. If so, as compared to the youngest age group, older children may be more likely to respond that the potential helpee would solve his problem on his own.

## MATERIALS AND METHODS

### PARTICIPANTS

The sample included fifteen 3.5-year-old children (3;4 years–3;11 years; 10 males), twenty 4.5-year-old children (4;7 years–4;8 years; 11 males), and sixteen 5.5-year-old children (5;6 years–5;11 years; nine males). Children were native German speakers from heterogeneous socioeconomic backgrounds. Informed consent for participation was given by the children’s caregivers. The participants received travel compensation and a small present for their participation. We followed the guidelines of the 1964 Declaration of Helsinki and the German Psychological Association.

### DESIGN AND PROCEDURE

**FIGURE 1:**
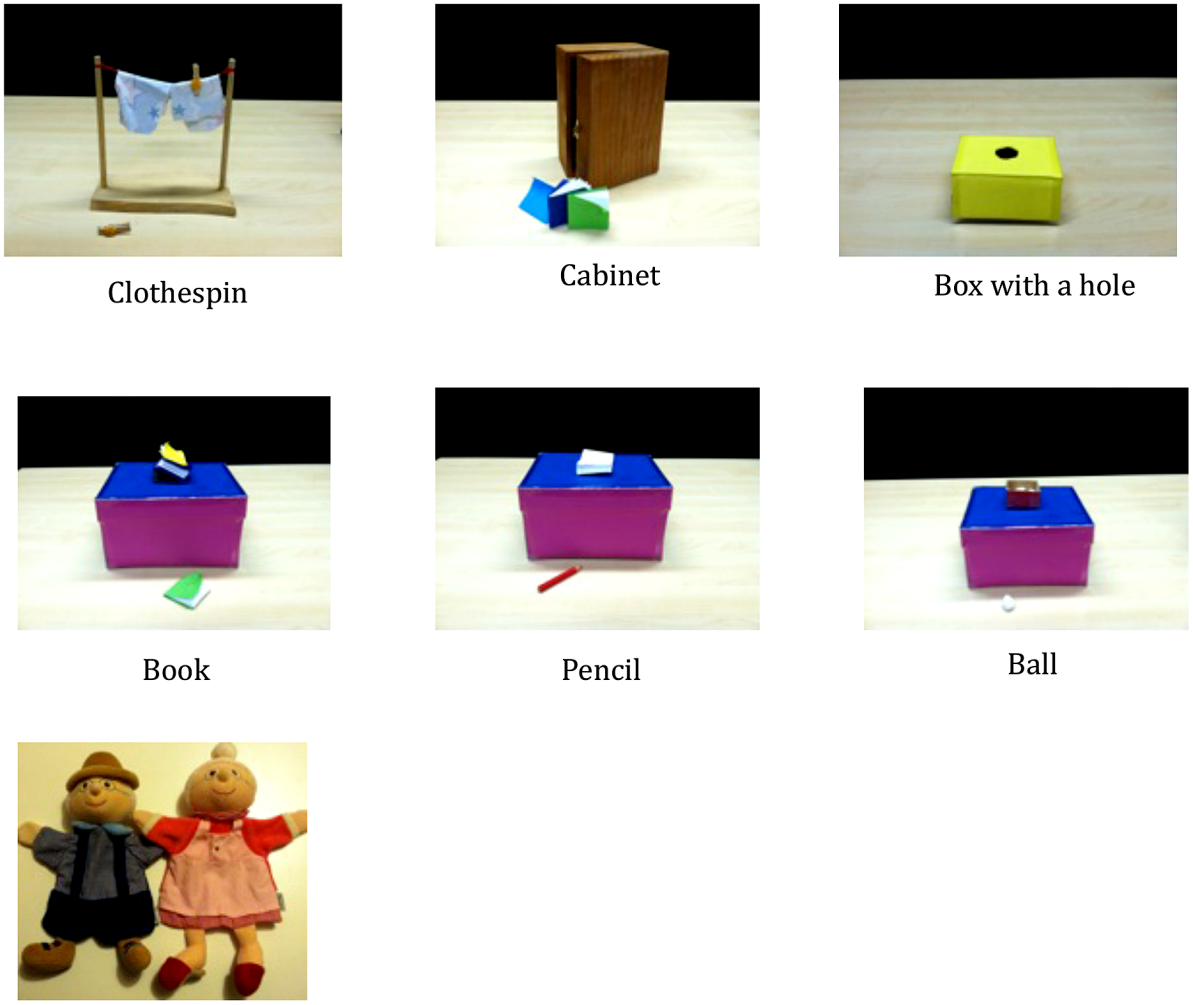
**Photograph of stimuli used in the study**.

Children were tested individually in a laboratory setting. Every child received a total of six tasks in one of the two predetermined (and thus pseudo-randomized) orders. In each task children saw two puppets (each operated by a different female experimenter) one of whom failed to complete his/her simple action goal (e.g., attempting to grab an object out of his/her reach) while the other was watching it and could offer help. For example, after the puppets greeted each other (“Hi”), one puppet indicated his intention to hang clothes on a clothesline, “Now I have to hang my clothes on a clothesline,” and successfully hung one piece of clothes on the clothesline with a clothespin. Then, as he hung another piece of clothes on the line, he accidentally dropped the clothespin on the floor and said, “Oops!” The puppet attempted to grab the clothespin out of his reach. He repeated his attempts to grab the clothespin but failed again. During the event, the other puppet was present without providing any remarks. See **Table 1** for an overview on six tasks and **Figure 1** for an overview on the stimuli used. Then, children were asked to predict what would happen immediately afterward (“How do you think the story should go on?”). If children did not respond for the first 10 s they were asked again, “Do you have any ideas what would happen next?” No child failed to respond. Children were also asked to justify their responses (e.g., “Why do you think she will pick up the clothespin?”). Children’s responses were videotaped and audio-recorded for the purpose of coding. Children saw the same pair of puppets across six tasks. Which of the two puppets served as a potential helpee was counterbalanced across the participants but fixed across tasks for any given child.

**Table 1:** A complete list of all the tasks used in the study.

Task	Problem
Clothespin	While hanging clothes on a clothesline, the puppet accidentally dropped a clothespin on a floor. He tried to grab the clothespin but failed.
Cabinet	While the puppet was putting books on the shelf in a cabinet, the cabinet door was accidently closed. He tried to open the door with his hands full of books but failed to open it.
Box with a hole	While carrying his favorite toy, the puppet accidentally dropped it into the hole in the box. The puppet tried to grab it by putting his hand into the hole but failed.
Book	While the puppet was stacking books on a table, one of the books slipped from the stack and fell on the floor. The puppet tried to grab the book but failed.
Pencil	While trying to draw a picture with a pencil, the puppet dropped the pencil on the floor. The puppet tried to grab the pencil but failed.
Ball	While putting a ball into a box, the puppet accidentally dropped it on the floor. The puppet tried to grab the ball but failed.

### CODING AND DATA ANALYSES

Children’s open-ended responses were coded into three main categories: *Self-action*: response indicating that the helpee would try (or manage) to complete the goal himself [e.g., “She (helpee) will pick up the clothespin”]; *Other-helping*: response indicating that the other puppet would offer help [e.g., “He (helper) will pick up the clothespin and give it to her”]; and* Other*: the remaining responses that did not fall into either one of the first two categories (e.g., “A bird will fly and take away the clothespin”). The coding categories were mutually exclusive; thus, none of the children’s response fell into more than one category. A second coder who was blind to the study hypotheses independently coded approximately 30% of the participants’ response randomly selected. Interrater reliability was 96% agreement; disagreements were resolved via discussion. We analyzed the number of trials (in percentages) in which children’s response fell into the *self-action*, the *other-helping*, and the *other* response. Children’s justifications were coded into two main categories: (1) *Desire:* response referring to the protagonist’s desire to fulfill the action (e.g., “He wants to draw the picture”); (2)* Capability*: response referring to the protagonist’s capability (e.g., “He can/cannot reach but she can/cannot”). There were unclassifiable statements (e.g., “Because the clothespin fell on the ground” or “So that he can say thank-you”) and no responses (e.g., “I don’t know”). Due to experimenter errors, 6 5.5-year-old children’s justifications were not asked. These children were excluded from the final analyses. A second coder independently coded the entire data. Interrater reliability was 90% agreement; disagreements were resolved via discussion.

## RESULTS

**FIGURE 2:**
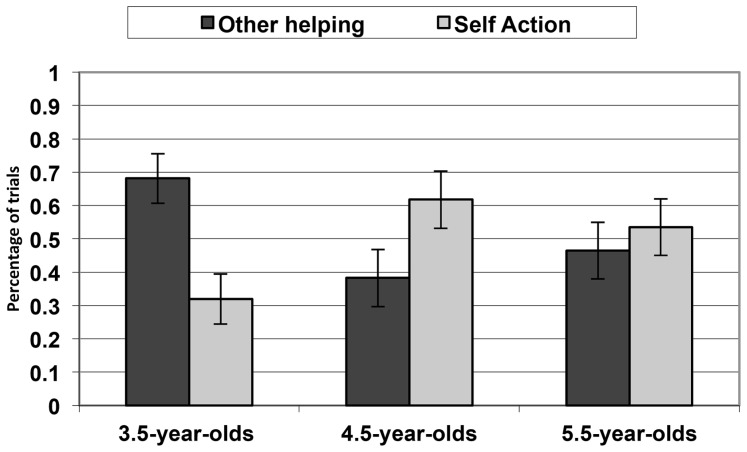
**Children’s expectations about others’ helping behaviors (as opposed to self-action) as a function of age groups.** The error bars indicate standard error.

Across age groups, children provided on average the other-helping response in 44.0% of the trials; the self-action response in 44.4% of the trials; and, other comments in 11.6% of the trials. For further analyses we omitted the other comments and focused on the self-action and other-helping responses. To this end, we calculated for every participant the percentages of the trials in which the other-helping responses were provided out of both response types. **Figure 2** presents the mean proportion of Other–helping response (as opposed to self-action) as a function of Age groups. Children’s responses of Other-helping were analyzed by means of a 2 (Gender: Male, Female) × 2 (Age Groups: 3.5, 4.5, 5.5) ANOVA with both variables as between subjects factors. There was only a significant effect of Age groups, *F*(2,45) = 4.182, *p* < 0.05, η^2^ = 0.16 (all other *p*s > 0.09).

3.5-year-old children provided the other-helping response more frequently than the 4.5-year-old children, *t*(33) = 2.512, *p* = 0.02. A similar trend was observed between 3.5- and 5.5-year-old children, *t*(29) = 1.902, *p* = 0.07. There was no difference between the 4.5- and 5.5-year-old children, *t*(34) = 0.668, *p* = 0.51.

The youngest age group of children tended to provide the other-helping response more frequently than the self-action response, *t*(14) = 2.426, *p* < 0.05. There was no significant effect for the 4.5- and 5.5-year-old children, *t*(19) = 1.362, *p* = 0.19, and *t*(15) = 0.416, *p* = 0.68, respectively.

**FIGURE 3:**
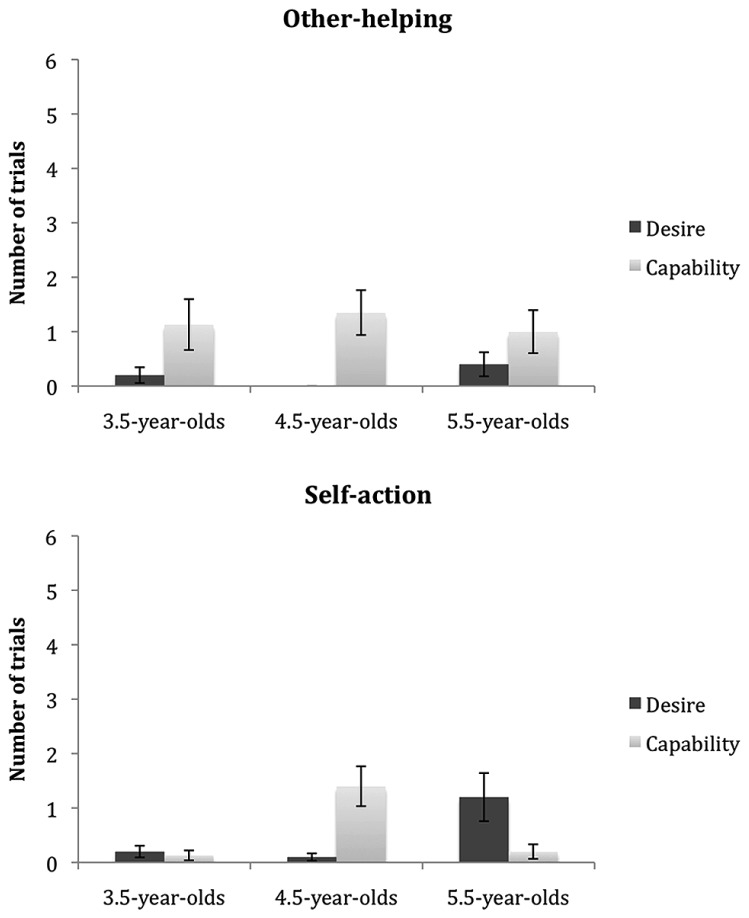
**The number of trials in which children’s justification fell to each category as a function of age groups and response types.** The error bars indicate standard error.

Next, we asked whether children’s justifications differed by the age groups and the response types. **Figure 3** presents the number of trials in which children’s justifications fell to each category (desire vs. capability) as a function of age groups and response types.

There was a trend among 3.5-year-olds to refer to capability more frequently than desire with respect to the other-helping response, *t*(14) = 1.86, *p* = 0.08, whereas their reference to desire and capability did not differ from one another with respect to the self-action response, *t*(14) = 0.44, *p* = 0.67. 4.5-year-old children referred to capability more frequently than desire both with respect to the self-action, *t*(19) = 3.51, *p* = 0.002 and the other-helping response, *t*(19) = 3.28, *p* = 0.004. There was a trend for 5.5-year old children to refer to desire more frequently than capability with respect to the self-action response, *t*(9) = 1.94, *p* = 0.08 whereas no significant difference was observed with respect to the other-helping response *t*(9) = 1.77, *p* = 0.11.

## DISCUSSION

The present research investigated young children’s expectation of others’ instrumental helping in a third party context. To this end, 3.5- to 5.5-year-old children were presented with the scenarios in which one person was in need of help in the presence of another person and were asked to complete the stories. As compared to 4.5- and 5.5-year-old children, 3.5-year-old children were more likely to expect another person to help someone who was in need of help. Moreover, with age children seem to consider different reasons for why one might or might not help someone. As compared to younger children, the oldest group of children equally referred to the characters’ desire and capability to complete an action related goal. These findings point to developmental changes in preschool children’s reasoning about others’ helping.

The present findings extend research on young children’s instrumental helping to young children’s reasoning about other people’s helping behavior. In particular, prior research showed that young children voluntarily helped someone complete goal directed actions (Warneken and Tomasello, 2006; Dunfield et al., 2011). In line with these findings, our results showed that 3.5-year-old children expected others to help another person who was in need of help. Note that in the present study children were not prompted by questions about helping. Instead, they were simply asked to predict what would happen in the stories. Nevertheless, the majority of 3.5-year-old children expected others to provide help to those who were in need of help. This suggests that by 3.5 years children have developed strong expectations about others’ helping.

How can we explain this finding? According to simulation theories of social cognition, people use their own behaviors and mental states to understand those of others (Goldman, 1989; Harris, 1989; Gallese and Goldman, 1998). Thus, 3.5-year-old children in the present research may rely on their own behavioral tendency to help others in order to predict others’ helping behaviors. Alternatively, they may detect regularities about others’ actions and use this information to predict future behaviors. Indeed, even 9-month-old infants expect others’ future action to be consistent with the most frequently performed action sequence in the past (Paulus et al., 2011). Children may be also able to detect the regularities of behaviors across different people. Additionally, it could be that the 3.5-year-old children are more likely to be helped by others than older children. Thus, 3.5-year-olds might have used prior experience and observation (e.g., a mother helping her child) to conclude that those in need of help are often likely to receive it from others.

Interestingly, as compared to 3.5-year-olds, older children displayed a different pattern of responses. Children of 4.5 and 5.5 years were equally likely to predict that the helpee would receive help or solve his problems on his own. One possible explanation for the age difference is that children’s ideas about, and underlying motives of, helping change during preschool years (see Hay and Cook, 2007). Older children may think that helping should be directed toward those who are indeed in need of help. Thus, whereas younger children provide help indiscriminately to others, older children may be selective in choosing who is or is not capable of solving one’s problems. Moreover, with age children may have a better understanding of a person’s capabilities in relation to the completion of his action goals. Indeed, Paulus and Moore (2011) demonstrated that preschool children’s understanding of others’ action capabilities develop between 2.5 and 5 years of age. Thus, it is plausible that as compared to 3.5-year-old children older children were more likely to reason that the protagonist’s action goals in the scenarios were within the range of his capabilities and thus he would not need help. Children’s justifications provide some support for this account. Older group of children, especially 4.5-year-old children, tended to refer to one’s capability to complete an action. Moreover, with age children’s justifications became more differentiated. The oldest group of children equally considered characters’ capabilities and desire to complete actions.

The present findings join a few recent studies (Sierksma et al., 2013; Paulus and Moore, 2014) in demonstrating that children hold a set of expectations about other people’s prosocial behaviors. Children expect others to share (Berndt, 1981) but more with friends than with disliked peers (Paulus and Moore, 2014). The present study showed that expectations of others’ instrumental helping are present in children as young as 3.5 years old.

Because one’s expectations of others’ social behaviors are closely related to evaluative behavioral judgments, the present findings have an implication for children’s moral and social judgments of others’ prosocial behaviors. In the present research, the youngest age group displayed the strongest expectation of others’ prosocial behaviors. This may be consistent with the findings that children of ages 2 and 3 years have strong expectations of others’ rule following (Rakoczy et al., 2008). With increasing age, however, children may become more lenient toward others’ lack of prosocial behaviors. Indeed, Sierksma et al. (2013) demonstrated that children of ages 8–13 years approved the refusal to help someone if helping was costly to the helper and the helpee’s need of help was low. Thus, it may be plausible that as compared to older children younger children may evaluate those who do not voluntarily offer help as more negatively. It is possible that children’s developing ideas of individual autonomy differentiated from their ideas about social and moral behavioral rules (e.g., Nucci and Turiel, 1978; Smetana et al., 1991; Smetana and Asquith, 1994) may also influence children’s reasoning about whether someone would receive help or independently solve his own problems.

Future research should address which principles and motives young children consider in reasoning about others’ instrumental helping. Specifically, children’s reasoning about different forms of helping needs to be further investigated. Although older children did not expect others to provide instrumental helping in the present study, it is possible that they may expect others to provide empathetic helping. Moreover, more research is needed to investigate whether and how children’s ideas about one’s autonomy in terms of action capabilities affect their own prosocial behaviors as well as reasoning about others’ prosocial behaviors. In general, how closely children’s developing ideas about others’ helping behaviors become related to their own helping behaviors, and what mechanisms support this relationship will improve our understanding of children’s prosociality.

## Conflict of Interest Statement

The authors declare that the research was conducted in the absence of any commercial or financial relationships that could be construed as a potential conflict of interest.
